# Functional Characterization of Argininosuccinate Lyase Gene Variants by Mini-Gene Splicing Assay

**DOI:** 10.3389/fgene.2019.00436

**Published:** 2019-05-17

**Authors:** Yanyun Wang, Yun Sun, Ming Liu, Xiaojuan Zhang, Tao Jiang

**Affiliations:** ^1^Center of Genetic Medicine, Women’s Hospital of Nanjing Medical University, Nanjing Maternity and Child Health Care Hospital, Nanjing, China; ^2^Center of Child Health Care, Women’s Hospital of Nanjing Medical University, Nanjing Maternity and Child Health Care Hospital, Nanjing, China; ^3^Center of Genetic Medicine, The Affiliated Obstetrics and Gynecology Hospital with Nanjing Medical University, Nanjing Maternity and Child Health Care Hospital, Nanjing, China

**Keywords:** argininosuccinate lyase (ASL), exon, intron, argininosuccinic aciduria (ASA), mini gene, aberrant splicing

## Abstract

**Objective:**

Argininosuccinate lyase (ASL) gene mutations account for argininosuccinic aciduria (ASA). This study aimed to design a minigene construct of ASL gene in order to investigate the impact of variants on splicing.

**Methods:**

The peripheral blood samples were collected from the family members, and genomic DNA was extracted for gene diagnosis using the total exon sequencing method. The novel mutation gene was cloned into pEGFP-C1 vector, and the pathogenicity of the mutation was examined in cultured cells *in vitro*.

**Results:**

The clinical diagnosis of the proband as ASA was clear. Two pathogenic mutations, c.281G>T (p.Arg94Leu) and c.208-15 T>A were detected in the ASL gene, and the two mutations had not been reported. The minigene expression *in vitro* confirmed that c.208-15 T>A could cause aberrant splicing, resulting in the retention of 13 bp in intron 2 to exon 3.

**Conclusion:**

Two new pathogenic mutations of ASL gene, c.208-15 T>A and c.281G>T, were found in an ASA family, which enriches the mutational profile of the ASL gene and provides a basis for genetic diagnosis of ASA. Minigenes are optimal approaches to determine whether the intron mutation can cause aberrant splicing.

## Introduction

Argininosuccinic aciduria (ASA) is a clinically rare urea cycle disorder, and belongs to the autosomal recessive genetic disease. ASA was first reported by ALLAN in 1958 (Allan et al., 1958). The overall reported prevalence of ASA is 1:70,000 (Nagamani et al., 2012c). ASA is due to the defect of argininosuccinate lyase (ASL) gene which causes arginyl succinic acid not to be cleaved into arginine and fumarate. The arginine succinic acid accumulates in a large amount in the body, and mainly manifests as hyperammonemia clinically. ASA can be divided into neonatal type and delayed type according to the onset of disease. Among them, the neonatal ASA is severe and the mortality rate is high (Chen et al., 2010). The first case of neonatal ASA in China was reported in 2014, and the diagnosis was confirmed by genetic testing after death (Yuan et al., 2014). Late-onset

type of ASA can occur in childhood or even in adulthood, and its manifestation is developmental delay and mental retardation. Baruteau et al. (2017) reported that the neurological phenotype of ASA patients did not correlate with the severity of hyperammonemia and plasma argininosuccinic acid levels. Currently, tandem mass spectrometry is the best method for early detection of ASA.

The ASL gene is located on 7q11.21 and has a total length of 23 kb with 17 exons (MIM: 608310), but the first exon encodes only for 5-untranslated region. Therefore, the coding region starts at exon 1 (NM_001024943.1) (Wen et al., 2016). Up to now, more than 60 ASL gene mutations have been reported, with high incidence in the exons 4, 5 and 7, and their mutation types are diverse, including nonsense mutation, missense mutation, insertion mutation, and deletion (Nagamani et al., 2012b).

However, in suspected patients, part of pathogenic gene variants are variants of unknown clinical significance (VUS), which makes genetic counseling of patients and their families complicated. More importantly, in families who wish to have a healthy child, this causes the difficulty in prenatal diagnosis. Therefore, functional studies for these variants are required. If the pathogenic mutation occurs in the intron, RNA from the patient should be examined by real time-polymerase chain reaction (RT-PCR) analysis to establish whether the variant has any effect on splicing. However, inherited metabolic diseases can manifest as rapid onset and rapid death, leading to the failure to timely keep samples for RNA extraction. Alternatively, the variant in intron can be examined by mini-gene splicing analysis (Bonnet et al., 2008; Théry et al., 2011). Here, we report two novel mutations in ASL gene in ASA patient, one of which is on the intron, and we functionally characterized the mutations.

## Materials and Methods

### Clinical Data

**FIGURE 1:**
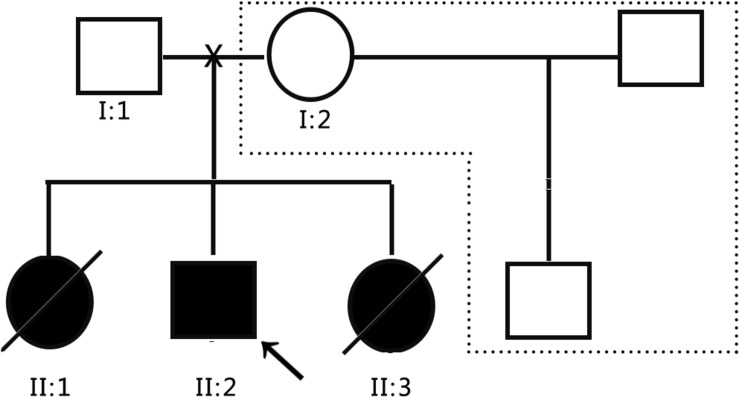
Schematic diagram of the proband pedigree.

The subjects were from the pedigree of Anhui Province, China. The medical history collection, biochemical tests, and genetic analysis were carried out in our hospital. The pedigree of the family is shown in Figure 1. The family is composed of only two generations and five members. Three persons still survive, and one of them is the patient. The proband (II: 2) is male and 8 years old. The patient’s father (I:1) and mother (I:2) are normal, three children were born, and the first child (II:1) is a daughter. When she was 10 years old, she suddenly fell into a coma without obvious incentives, and died within 24 h. Her blood ammonia > 1,000 μmol/L, but related medical data have been lost. The third child (II: 3) is a daughter. When she was 3 years old, she suddenly fell into a coma without obvious incentives, and was sent to the hospital for rescue and died within 3 days. During the treatment, Tandem mass spectrometry (MS/MS) showed that citrulline (CIT) increased to 100 μmol/L, and blood ammonia was >1,000 μmol/L, considering the possibility of inherited metabolic diseases, but the specimen was not timely kept for genetic testing. In view of the condition of II:1 and II:3, the patient’s parents gave the proband (II: 2) almost pure vegetarian feeding, and he survives till today, but gradually showed hot temper and mental retardation, and is unable to be enrolled in school for normal education. In order to obtain a clear diagnosis and give birth to a healthy child, the patient took II:2 to the specialist clinic of inherited metabolic diseases of our hospital in 2016. After the patient’s parents signed the informed consent form, 2 ml peripheral venous blood was drawn from everyone to extract genome DNA. MS/MS and blood ammonia were rechecked, blood ammonia was 200 μmol/L, and CIT in dry blood spot was 118.65 μmol/L detected by MS/MS, and kidney functional electrolytes were normal. This pedigree has only DNA samples of three persons: the parents of the proband and the proband.

### Total Exon Detection

2 ml venous blood samples were collected from the proband and his parents. Genomic DNA (QIAamp DNA Blood Midi Kit, Qiagen, Hilden, Germany) was extracted according to the standard procedure and sent to Shenzhen Huada Gene Clinical Inspection Center for total exon detection. The DNA fragments were sequenced using a high-throughput sequencer Illumina HiSeq 2500 Analyzers (Illumina, San Diego, CA, United States), and raw sequencing data was read using Illumina Pipeline software (version 1.3.4). Sequence alignment with HG19 was performed using BWA (Burrows Wheeler Aligner) software. The SNV (single nucleotide variant) and Indel (insertion and deletion) queries were performed using SOAPsnp software and Samtools software, respectively, to generate target region base polymorphism results, followed by the comparison of database (NCBI dbSNP, HapMap, 1000 human genome dataset and database of 100 Chinese healthy adults), the suspicious mutations were annotated and screened. For all pathogenic mutations, the primers were designed upstream and downstream of the fragment in which they were located. Polymerase chain reaction (PCR) amplification was performed, and Sanger sequencing was performed on PCR product to verify the results of gene chip capture and high throughput sequencing.

### *In silico* Analysis

The bioinformatic splicing tool HSF (Human Splicing Finder) version 3.0^1^ was applied to predict the possible influence of mutation in intron.

### Construction of Recombinant Plasmids

**FIGURE 2:**
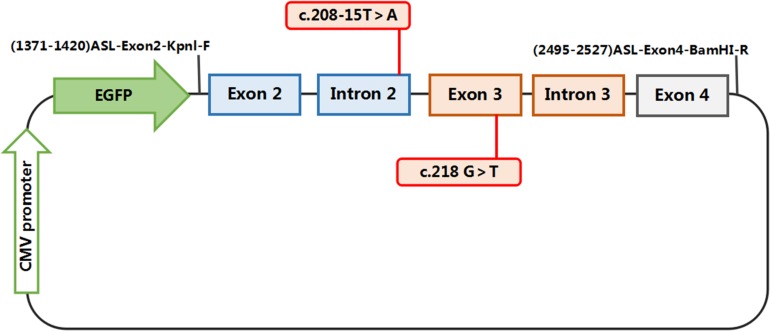
Structures and functional analysis of the splicing vector pEGFP-C1 and minigene ASL-Wt /ASL-Int-mut (C.208-15 T > A)/ASL-Exon-mut (C.281 G > T): [EX2 – IVS2 – EX3 – IVS3 – EX4]: the pEGFP-C1 vector contains a CMV promoter. The expected splicing reactions in eukaryotic cells are indicated by arrows.

The constructs were made by three consecutive rounds of PCR: the first PCR was performed using genomic DNA (a total of three sets of DNA) as a template, and ASL-176-F and ASL-1758-R as primers, 30 cycles; the secondary PCR was performed using the first round of PCR products as a template, and ASL-448-F and ASL-1674-R as primers, 30 cycles; the third PCR was performed using PCR product of the second round as a template, and ASL-KpnI-F and ASL-BamHI-R as primers, 30 cycles (primers were shown in Tables 1, 2). The electrophoresis and gel recovery of the last round PCR product was performed. ASL-Wt (Wild-type), ASL-Int-mut (c.208-15 T > A), and ASL-Exon-mut (c.281 G > T) all contained the entire sequence of exon 2 to exon 4, the amplified length was 1,157 bp, and the amplified gene region was shown in Figure 2.

**TABLE 1:** Genomic DNA and gene names corresponding to each group.

**Genomic DNA**	**Name of gene**
(1) Control group	ASL-Wt (Wild-type)
(2) Experimental group (proband)	ASL-Int-mut (c.208-15 T > A)
(3) Experimental group (proband’s father)	ASL-Exon-mut (c.281 G > T)

**TABLE 2:** Primersused in this study.

**Primers**	**Sequence 5′→3′**	**Melts degree**	**Product length**
ASL-176-F	cagcctggccgacaaagtgagac	58	1583
ASL-1758-R	ggcccgagagcgtggagcaggtc	65	
ASL-448-F	ggccggtttgtgggtgcagtg	61	1227
ASL-1674-R	ggccgatgtgttgcttccctgag	60	
ASL-KpnI-F	cgacggtaccatgagtgggaagctt tggggtggccggtttgtgggtgcag	62	1157
ASL-BamHI-R	cggtggatccctggtca ttccggctccgtcccg	62	

Polymerase chain reaction (PCR) products were purified, ASL-Wt (Wild-type), ASL-Int-mut (c.208-15 T > A) and ASL-Exon-mut (c.281 G > T), and inserted into the eukaryotic expression vector pEGFP-C1 using KpnI/BamHI to construct three sets of plasmids: pEGFP-C1-ASL-wt, pEGFP-C1-ASL-Exon-mut, and pEGFP-C1-ASL-Int-mut. The recombinant plasmids were digested with KpnI and BamHI, and verified by gene sequencing.

### Transfection of Eukaryotic Cells

The recombinant vectors (pEGFP-C1-ASL-wt, pEGFP-C1-ASL-Exon-mut, and pEGFP-C1-ASL-Int-mut) were transiently transfected into human lung epithelial cells (A549), human embryonic kidney cells (HEK-293T) and cervical cancer cells (Hela) according to the instructions, and the transfected cells were cultured for 48 h and then collected for analysis.

### Real Time-Polymerase Chain Reaction

The total RNA was extracted from A549, 293T, and HeLa cells by Trizol method (RNA extraction kit, Omega), and the cDNA was reverse transcribed using a reverse transcription kit (Thermo) according to the manufacturer’s instructions. The concentration and purity of the extracted RNA were determined by UV spectrophotometry. PCR products were identified by 2% agarose gel electrophoresis and verified by sequencing.

## Results

### Genetic Test Results

**FIGURE 3:**
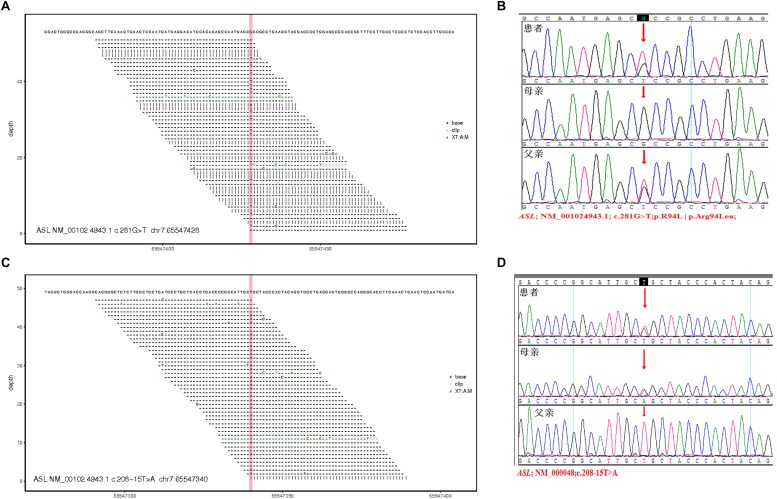
Total exon sequencing map and Sanger sequencing map: two pathogenic mutations C.281G > T (P.R94L|p.Arg94Leu) and C.208-15 T > A are detected in the ASL gene, and they have not been reported. **(A,C)** Total exon sequencing map. **(B,D)** Sanger sequencing map for pedigree verification, C.281G > T(P.R94L|p.Arg94Leu) is inherited from the patient’s father; C.208-15 T > A is inherited from the patient’s mother.

Two pathogenic mutations were detected in the ASL gene, as shown in Figure 3, Table 3, and Supplementary Table S1. No other disease-causing mutations were detected in the pathogenic genes of diseases that caused an increase in CIT. The parents of this patient divorced when the patient was diagnosed by genetic test, and the mother (I: 2) re-establishes a new family, and has a healthy baby boy. Now the patient and his father only received follow-up in peripheral blood examination by MS/MS and did not like to provide sample for RNA extraction. Alternatively, the variant in intron can be examined by mini-gene splicing analysis. We constructed a mini-gene vector for the new mutation of c.208-15 T > A, and further confirmed whether the mutation would cause aberrant splicing of mRNA and cause abnormalities in gene expression.

**TABLE 3:** Total exon detection results.

**Gene**	**Reference sequence**	**Nucleotide change/ mutation name**	**Amino acid change**	**Gene subregion**	**Heterozygosity**	**Chromosome location**	**Reference**	**Variant type**	**Inherited**
ASL	NM_ 001024943.1	c.281G > T	p.Arg94Leu	EX3	Heterozygous	Chr:65547428	–	VUS	Father
ASL	NM_ 000048	c.208-15 T > A	–	–	Heterozygous	Chr:65547340	–	VUS	Mother

### *In silico* Splicing Analysis

Human Splicing Finder can be used to identify splicing mutations, providing a better understanding of clinical and biological data. Here, this bioinformatic tool was applied and showed that the mutation of c.208-15T > A in Intron is an “Activation of an intronic cryptic acceptor site,” and if cryptic site use, exon length will increase 13 bp.

### Construction of Recombinant Plasmids

**FIGURE 4:**
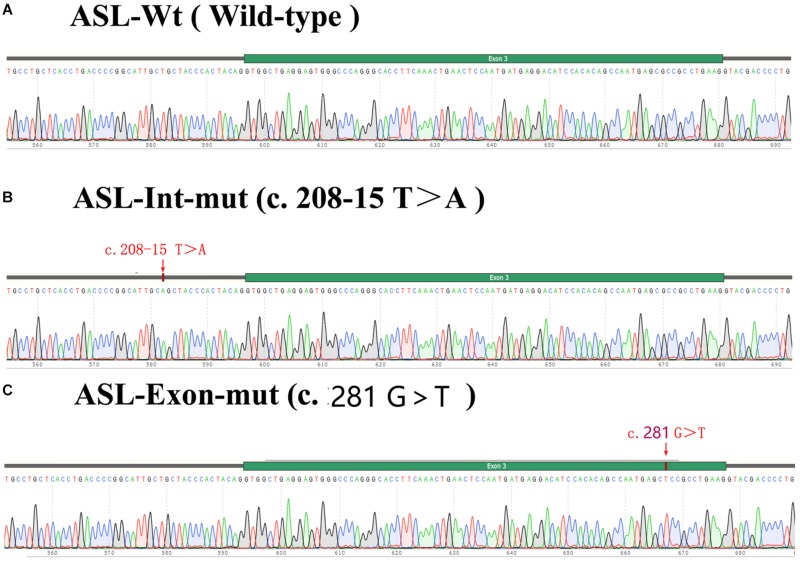
Sequencing results of target fragment **(A–C)**ASL-wt/Exon-mut/Int-mut minigene.

The exon 2 to exon 4 of the ASL gene of the proband and his father were amplified by PCR and recombinnat plasmids pEGFP-C1-ASL-wt, pEGFP-C1-ASL-Exon-mut, and pEGFP-C1-ASL-Int-mut were successfully constructed. The three recombinant plasmids were digested with restriction endonuclease KpnI and BamHI, and two fragments were found to be consistent with the expected ones, confirming that the target fragment ASL-wt/Exon-mut/Int-mut minigene was successfully inserted into the vector. The sequencing results are shown in Figure 4.

### ASL mRNA Expression in Cells Transfected With Recombinant Plasmids

**FIGURE 5:**
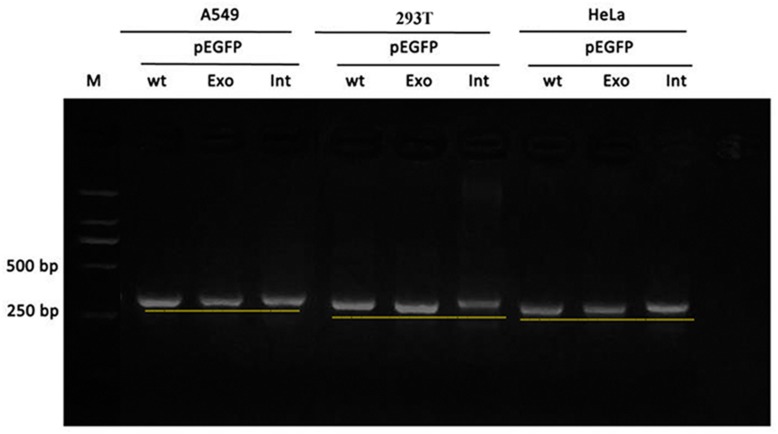
Gel electrophoresis of RT-PCR products: the band of the intron mutant (Int) was bigger than the wild-type (wt) and the exon mutant (Exon).

**FIGURE 6:**
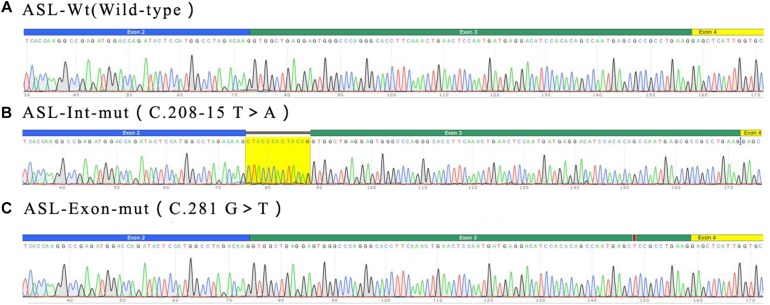
Minigene product sequencing results: **(A)** wild-type minigene (ASL-wt) formed normal mRNA composed of exons 2, 3, and 4; **(B)** the intron C.208-15 T > A mutant minigene caused a splicing abnormality, resulting in the retention of the 13 bp base in intron 2; **(C)** the exon mutant minigene (ASL-Exon) did not affect splicing, and its splicing pattern is consistent with the wild-type **(A)**.

**FIGURE 7:**
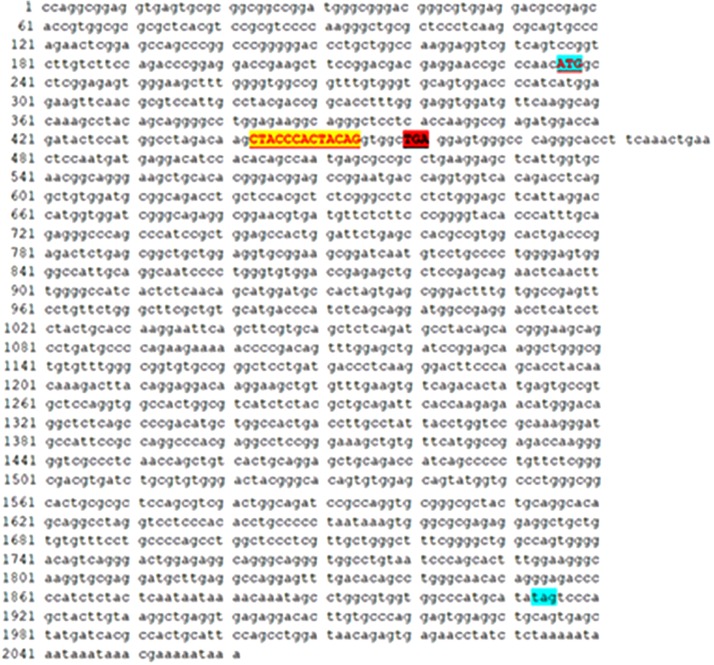
The intron c.208-15 T > A mutant minigene caused aberrant splicing, resulting in the retention of the 13 bp base in intron 2 to exon 3. There are 222 codons (74 amino acid residues) between the promoter (ATG) and the first termination codon (TGA) because of the insertion of 13 bases. In theory, there are 1596 codons (532 amino acid residues) between the promoter (ATG) and the first termination codon (TAG) in ASL gene.

Real time-polymerase chain reaction analysis of ASL gene showed that the band of the intron mutant (Int) was bigger than the wild-type (wt) and the exon mutant (Exon) and the migration became slower (Figure 5). DNA sequencing results (Figure 6) indicated that wild-type minigene (ASL-wt) formed normal mRNA composed of exons 2, 3, and 4; the exon mutant minigene (ASL-Exon) did not affect splicing, and its splicing pattern is consistent with wild-type. The intron c.208-15 T > A mutant minigene caused aberrant splicing, resulting in the retention of the 13 bp base in intron 2 to exon 3 (Figures 6, 7). This result is consistent with HSF analysis result.

## Discussion

Two mutations in the ASL gene of the proband in this study are detected: (1) exon 3: c.281G > T (p.Arg94Leu) from the father; (2) intron 2: c.208-15T > A from the mother. Two suspicious pathogenic mutations have not been reported, of which c.281G > T is a missense mutation, the incidence is extremely low in the population, and SIFT and PolyPhen software predicted it to be a pathogenic mutation. c.208-15T > A is an intron region mutation, the incidence in population is low, and the clinical significance is unknown. According to the “Interpretation Criteria and Guidelines for Gene Sequence Variation” established in 2015 (Richards et al., 2015), the pathogenicity analysis is carried out: (1) the incidence of two mutation sites in the normal population is very low, does not belong to polymorphic change (medium strong pathogenic evidence PM2); (2) the two pathogenic sites are both on the ASL gene, are derived from his father and mother, respectively, and are consistent with the hereditary rule of recessive hereditary disease (moderate strong pathogenic evidence, PM3); (3) before the death, the MS/MS test results of 2 sisters of the patient are similar to that of the patient, both show an increase in CIT accompanied by hyperammonemia, the clinical manifestations of the patient and similar manifestations of family members are highly consistent with the phenotype of arginyl succinate induced by ASL gene mutation (supportive pathogenic evidence, PP4). Taken together, the evidence intensity of c.281G > T and c.208-15T > A mutation is “PM2+PM3+PP4” and is judged to be a pathogenic mutation (very strong pathogenic evidence).

In order to further clarify the pathogenicity of suspicious intron mutations, we performed RT-PCR splicing validation by constructing the miningene vector. The results showed that the intron c.208-15 T > A mutation caused aberrant splicing, resulting in the retention of 13 bp in intron 2 to exon 3.

The clinical interpretation of the splicing results of a genetic variant is complex. A variant would be considered pathogenic when it causes frame shift, major splicing aberrations and in-frame insertion/deletion. In HSF and mini-gene, the variant c.208-15T > A was predicted to cause in-frame insertion of 13 bp (Supplementary Figure S1). Normally, there are 1596 codons (532 amino acid residues) between initiation codon (ATG) and the first termination codon (TAG) in ASL gene. However, there are 222 codons (74 amino acid residues) between initiation codon (ATG) and the first termination codon (TGA) because of the insertion of 13 bases, and therefore the deletion of 458 amino acid residues could change the structure and function of the protein. Thus the mutation site may be one of the pathogenic mutations of the proband.

The exon c.281G > T mutation does not affect the splicing, and the splicing pattern is consistent with wild-type, but the mutation is from the father, causing the change of amino acids. Its incidence is extremely low in the population, and SIFT and PolyPhen software predicted it to be a pathogenic mutation so we tend to think that this site is also the pathogenic site of ASA.

Effective treatment is very important for ASA patients. The drug treatment with arginine, sodium benzoate, and lactulose can reduce ammonia. In the remission period there is no specific drug and low-protein and high-calorie diet, mainly starch carbohydrates such as rice and noodles, can reduce the protein decomposition in the body. It is speculated that the proband in this study survives because of the long-term low-protein diet, but his quality of life is poor, with intelligence and mental development abnormality. In addition, the patient can be treated with nitrogen scavenger sodium phenylbutyrate orally, supplemented with l-carnitine and arginine, and avoid the ingestion of sodium valproate and hepatotoxic drugs (Uçar et al., 2014). Fagerberg et al. examined the distribution of ASL enzyme, based on RNA sequencing of 95 normal tissue samples representing 27 different tissues, it was found that ASL was mainly present in the liver (Fagerberg et al., 2014), which provides a basis for the treatment by liver transplantation. The long-term prognosis for patients with ASA under pharmacologic and dietary therapy remains poor. Liver transplant should be considered a treatment option in selected cases (Mercimek-Mahmutoglu et al., 2010; Nagamani et al., 2012a; Yankol et al., 2016). Liver transplant provides sufficient enzymatic activity to correct the deficiency, and reduces the risk of metabolic decompensation with dietary protein restriction. However, liver transplant in neonates or small infants (under 1 year of age) is still technically challenging and has a high morbidity and mortality rate.

In summary, based on the minigene vector technology, we confirm that the intron c.208-15 T > A mutation can cause aberrant splicing, resulting in the retention of 13 bp in intron 2, and is a pathogenic site. Splicing reporter minigenes in our study have the following advantages: (i) no need to extract RNA from the patient; (ii) analysis and quantification of the splicing outcome of a single mutant allele without the interference of the wild-type one; (iii) high reproducibility of results. Our reports on c.208-15 T > A and c.281G > T pathogenic sites enrich the mutational profile of the ASL gene and provide a basis for genetic diagnosis of ASA.

## Ethics Statement

This study was approved by the local Ethics Committee of Nanjing Maternity and Child Health Care Hospital. Informed written consent was obtained from all patients prior to their enrollment in this study. We sincerely thank all the family members for their participation and cooperation in this study.

## Author Contributions

YW conceptualized and designed the study, completed the experiment, led the review process, and drafted the initial manuscript. YS reviewed all manuscript. XZ assisted YW to complete the experiments. ML responsible for the physical examination and follow-up of children. TJ responsible for the overall content. All authors made substantial contributions to revising the manuscript and read and approved the final manuscript.

## Conflict of Interest Statement

The authors declare that the research was conducted in the absence of any commercial or financial relationships that could be construed as a potential conflict of interest.

## Footnotes

1 http://www.umd.be/HSF3/

## Supplementary Material

FIGURE S1 *In silico* predictions for c.208-15T > A in intron using HSF.

TABLE S1 Verification results of mutation sites in pedigree.
