# Supplementary figures and images for: Functional Characterization of Argininosuccinate Lyase Gene Variants by Mini-Gene Splicing Assay
Source: Front Genet. 2019 May 17;10:436. doi: 10.3389/fgene.2019.00436 (PMC6533879; doi:10.3389/fgene.2019.00436)

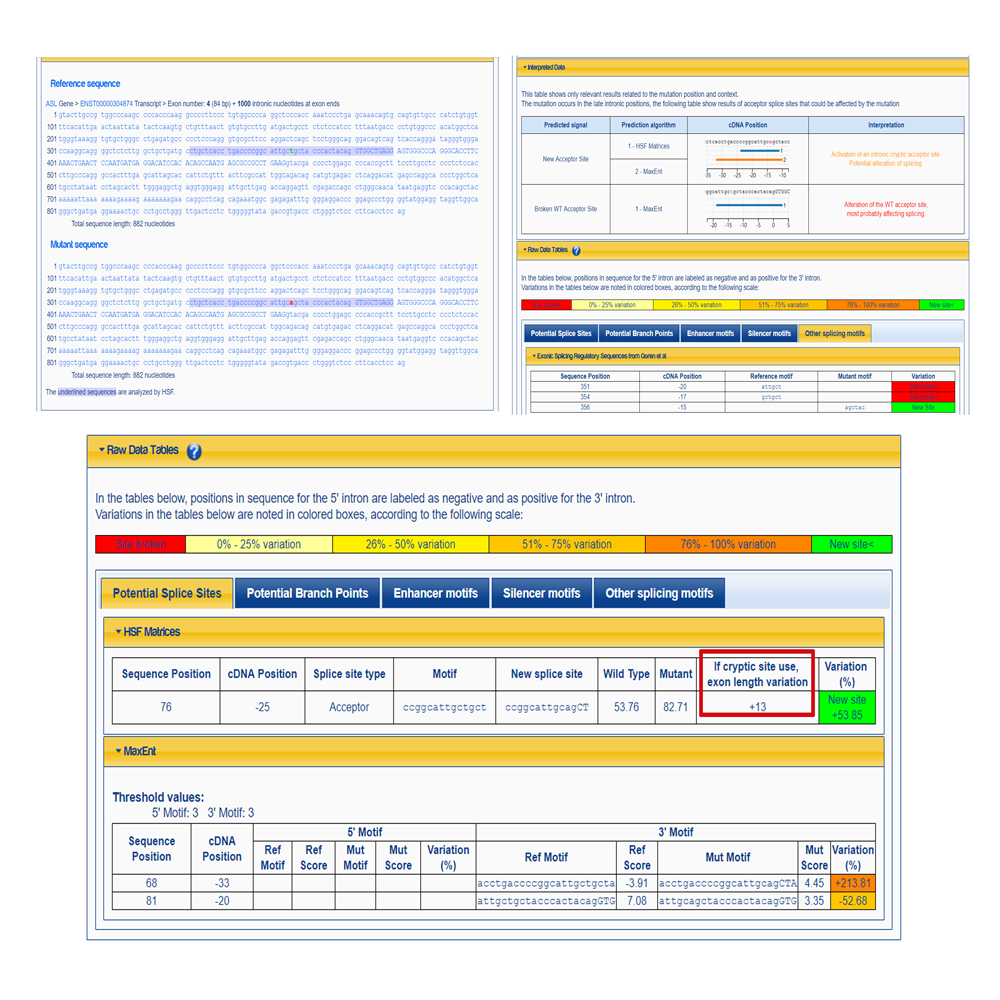

Supplement: FIGURE S1 — In silico predictions for c.208-15T > A in intron using HSF. [file Image_1.TIF]
