# Supplementary material for: Functional Characterization of Argininosuccinate Lyase Gene Variants by Mini-Gene Splicing Assay
Source: Front Genet. 2019 May 17;10:436. doi: 10.3389/fgene.2019.00436 (PMC6533879; doi:10.3389/fgene.2019.00436)
Supplement: TABLE S1 — Verification results of mutation sites in pedigree. [file Table_1.docx]

gttcaagaccagcctggccaacatggtgaaaccccatctctactaatgatacaaaaattagctgggtgtggtggcacgtgcctgtaatcccagctacttgggagtctgaggcaggagaatctcttgaacctgggaggcagagtttgcagtgagctgagatggcaccactgtactccagcctggccgacaaagtgagactctgtctcaaaaaaaaaaaaaaagaacccaaacttttggtgttcagccatgttcccatgctcactcccagggtggtgactctgggaaggtctcagcctccttgtctgcccagttagaatgatctgatgcccctgctaccatcagacttgataagtttcccaaagactctttgcaagaagcactgttctggagggtggaggagagactaattgttcttgctctcctggccagagtgggaagctttggggtggccggtttgtgggtgcagtggaccccatcatggagaagttcaacgcgtccattgcctacgaccggcacctttgggaggtggatgttcaaggcagcaaagcctacagcaggggcctggagaaggcagggctcctcaccaaggccgagatggaccagatactccatggcctagacaaggtacttgccgtggcccaagccccacccaaggccccttccctgtggccccaggctcccaccaaatccctgagcaaacagtgcagtgttgcccatctgtggtttcacattgaactaattatatactcaagtgctgtttaactgtgtgccttgatgactgcctctctccatcctttaatgacccctgtggcccacatggctcatgggtaaaggtgtgctgggcctgagatgccccctcccagggtgcgcttccaggactcagctcctgggcagggacagtcagtcaccagggatagggtgggaccaaggcaggggctctcttggctgctgatgcctgctcacctgaccccggcattgc**t**gctacccactacaggtggctgaggagtgggcccagggcaccttcaaactgaactccaatgatgaggacatccacacagccaatgagcgccgcctgaaggtacgacccctggagccccaccgctttccttgcctcccctctccaccttgcccagggccactttgagcattagcaccattctgtttacttcgccattggcagacagcatgtgagacctcaggacatgagccaggcaccctggctcatgcctataatcctagcactttgggaggctgaggtgggaggattgcttgagaccaggagttcgagaccagcctgggcaacataatgaggtcccacagctacaaaaattaaaaaaagaaaagaaaaaaagaacaggcctcagcagaaatggcgagagatttggggaggacccggagccctggggtatggaggtaggttggcagggctgatgaggaaaactgccctgcctgggttgactcctctgggggtatagaccgtgaccctgggtctcccttcacctccaggagctcattggtgcaacggcagggaagctgcacacgggacggagccggaatgaccaggtgctttagcccctccaccccctgctccgtgttgtcccaaccttgaggagcccagggggcagttagagttctgcagcggtcctggctcctcagggaagcaacacatcggcctccctgagcaccatctcctccttgcacaggtggtcacagacctcaggctgtggatgcggcagacctgctccacgctctcgggcctcctctgggagctcattaggaccatggtggatcgggcagaggcgtgagtcctacagggacacccagggggcagacagaggtgtgatggaagcctgaacaggagacctagggggcaggggtgaacagcgtgggggtgccaggccctgggggacaggggcatcccagaactccaggatcgaggcagagcagccaggagtgggccatttcctgcaggccccaatactcccatgccagtctagctcagcaggcagagaagactaacccttcgtggggctgggtgcggtggctcacgcctgtaatctcagcactttgggaggccgaggtgggtggatcacctgaggtcaggagttcgagaccagcctggccaacatgggaaaactctgtctctactaaaaatacaaaaattaggcaatgtggtggtgtgcgcctgtaatcccagctactcgggagcctgaggcagaagaactgcttgaacccgggaggaggaggttgcaatgagccgagatcgcgccattgcactccagcctgggctacagagcgagactcctgtctcaaaaaaaaagaaaaaaaaaaaagaaaactcaccatttgcagatttgaaggcaggaagctaagccaagcacagctagcttggctgtgcctggagcagccagagtcactccccacactgcctgtcccccagatcccccatcctaagcttcgcctccccatccagcccatctggcaaaagacagagccaaag

ASL-176-F: 5' cagcctggccgacaaagtgagac 3' melts at: 58 degrees

ASL-1758-R: 5' ggcccgagagcgtggagcaggtc 3' melts at: 65 degrees

Product Length: 1583

ASL-448-F: 5' ggccggtttgtgggtgcagtg 3' melts at: 61 degrees

ASL-1674-R: 5' ggccgatgtgttgcttccctgag 3' melts at: 60 degrees

Product Length: 1227

ASL-521-F: 5' gggaggtggatgttcaaggcagca 3' melts at: 62 degrees

ASL-1131-R: 5' gggcaaggtggagaggggaggc 3' melts at: 62 degrees

Product Length: 611

| ASL-Exon2-KpnI-F | CGACGGTACCatgagtgggaagctttggggtggccggtttgtgggtgcag |
| --- | --- |
| ASL-Exon4-BamHI-R | CGGTGGATCCctggtcattccggctccgtcccg |

Product Length: 1157
